# The Lazarus Phenomenon Among Older People—A Descriptive Analysis of Cases Spanning over 40 Years

**DOI:** 10.3390/jcm15134855

**Published:** 2026-06-23

**Authors:** Małgorzata Grześkowiak, Anna Kluzik, Piotr Rzeźniczek, Agnieszka Danuta Gaczkowska

**Affiliations:** 1 Department of Anaesthesiology and Intensive Therapy Teaching, Poznań University of Medical Sciences, Święcickiego St. 6, 60-781 Poznań, Poland; mgrzesko@ump.edu.pl (M.G.); przezniczek@ump.edu.pl (P.R.); agaczkowska@ump.edu.pl (A.D.G.); 2 Department of Anaesthesiology, Intensive Therapy and Pain Treatment, Poznań University of Medical Sciences, Grunwaldzka St. 55, 60-352 Poznań, Poland

**Keywords:** Lazarus phenomenon, Lazarus syndrome, auto-resuscitation, cardiopulmonary resuscitation (CPR), elder patients, geriatric

## Abstract

The Lazarus phenomenon (LP), also called auto-resuscitation, may happen after the end of ineffective cardiopulmonary resuscitation (CPR), or after death is confirmed in a person who did not undergo CPR, and heart activity returns spontaneously. The aim of the study was to focus on older individuals (aged >60) experiencing the LP and to analyse distractors that cause this phenomenon. **Methods.** PubMed, Scopus, and Web of Science electronic databases were searched to find cases of LP from the year 1982 until 31 December 2025. Of the 81 total cases found, 48 patients were included in the study. For the analysis they were divided into two subgroups dependent on age: No 1 (60–79), No 2 (≥80). **Results.** Based on the descriptive analysis, the causes of cardiac arrest were divided almost equally between cardiac and non-cardiac causes (47.6% and 52.3% respectively). Cardiac arrest occurred equally in the IH and OH. In 16 out of 37 cases where such data were reported, a return to consciousness was confirmed, representing 43.2%. **Conclusions.** In older people, even those of very advanced age, the Lazarus phenomenon may occur. Based on the analysis carried out and given the lack of available data and the small sample size (48 individuals), it is not possible at this stage to definitively identify the causes of LP in the elderly population. As a potential cause of LP, age-related changes should be taken into account. Given that LP also occurs in the older population, consideration should be given to the need for extended monitoring of vital signs following the declaration of death. With a view to raising awareness of LP, it seems appropriate to include information on this phenomenon in the CPR guidelines.

## 1. Introduction

The Lazarus phenomenon (LP) is rare and can occur in people of all ages. Unfortunately, its causes are still unknown. This phenomenon consists of the fact that either after the end of ineffective cardiopulmonary resuscitation (CPR), heart activity returns spontaneously, or after death is confirmed in a person who did not undergo CPR, heart activity returns. The commonly used synonym is auto-resuscitation. For the first time in the scientific literature, in 1982, K. Linko et al. described the recovery after discontinued cardiopulmonary resuscitation for cardiac reasons in a 67-year-old man and 68-year-old woman [1]. In 1993 Bray JG Jr. used the term “Lazarus phenomenon” [2] for auto-resuscitation, which means a phenomenon of the heart that can resume its spontaneous activity and generate circulation [3].

At the current stage of knowledge, the causes of the Lazarus phenomenon are only presumed. The following reasons are considered: hyperventilation, alkalosis, auto-PEEP (positive end expiratory pressure), delayed drug action, hyperkalaemia, unnoticeable vital signs and metabolic disorders [4,5]. In hyperventilation, expiratory time is shortened, resulting in increased intrathoracic pressure and decreased venous return, leading to decreased cardiac output. This disorder additionally explains a slower delivery of drugs to the central circulation and finally delayed action of drugs, which is given as the other explanation for the Lazarus phenomenon. After stopping CPR in a hyperventilated patient, their state can be changed and even reversed—the pressure in the chest is lower than during the hyperventilation period and this mechanism may be responsible for restart of the heart. Auto-PEEP, positive end expiratory pressure, can build up in the airway and similarly to hyperventilation may lead to an increase in intrathoracic pressure. By the same mechanism that hyperventilation can cause, auto-PEEP may impair venous return to the heart and decrease cardiac output [6,7]. It should also be emphasised that many authors trying to explain the causes of Lazarus phenomenon indicated the coexistence of many diseases in these patients such as: cancer, cardiovascular diseases (cardiomyopathy), ischemic heart disease, sepsis and surprisingly advanced age [8,9,10].

The Lazarus phenomenon is not a real disease itself, but a phenomenon of the body that can happen to anyone. Because occurrence of LP is very rare, only 81 cases of the Lazarus phenomenon over the course of 40 years were published in the scientific literature. Updated case studies on LP have been published in recent years by David J. Zorko et al. in 2023 [11] and later by M. Grześkowiak and P. Rzeźniczek [12,13]. In 2023–2025 five more cases were published and included in the study. In terms of the ageing population, we noticed that LP occurred quite frequently in older patients (total 81 cases, 48 cases > 60 years of age, 59.25%), hence the aim of the study was to focus on older individuals experiencing the Lazarus phenomenon and to analyse distractors that cause the LP in these patients, with particular emphasis on two age subgroups.

## 2. The Methods

As the population ages, the number of older people is increasing, which is why we have focused our analysis on this group. There is no single, definitive definition of old age. It is highly fluid, and the age limit is conventional. For research purposes, the demo-graphic onset of old age is most often assumed to be 60 years (WHO). A uniform age of older age is used in studies for women and men. Further subcategories have been proposed within this population: advanced age, also known as early old age (young old), old age, also known as late old age (old-old), and advanced old age, also known as longevity. For the purposes of this study, we expanded the definition of early old age to 80 years and grouped the subsequent categories into a single group, ≥80 years, to facilitate analysis.

The subsequent stages are early old age, late old age and advanced old age. We have extended the definition of early old age by five years to 80, and have grouped the subsequent stages together.

**Figure 1:**
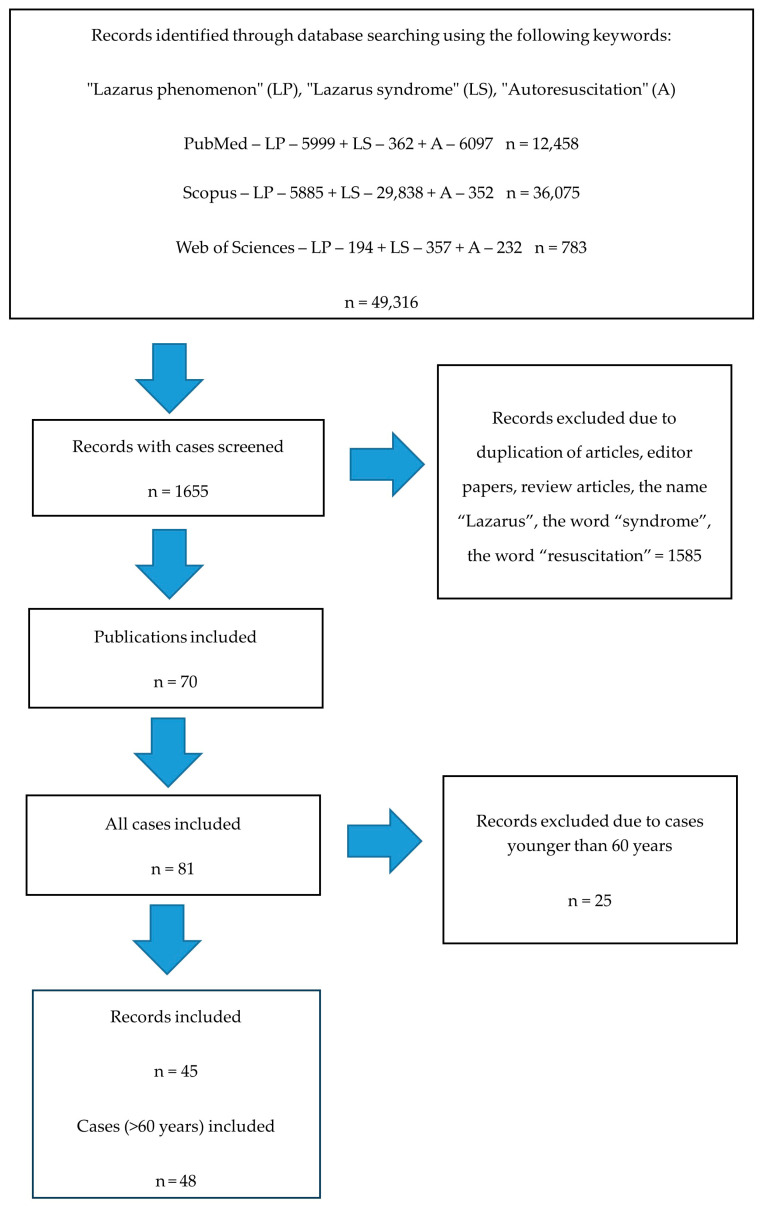
Flowchart of case selection based on PRISMA.

Based on PRISMA we have searched PubMed, Scopus, and Web of Science electronic databases containing all reports published from the year 1982 until 31 December 2025 looking for the phrases: “Lazarus phenomenon” or “Lazarus or phenomenon”, “Lazarus syndrome or Lazarus or syndrome”, and “Autoresuscitation or Autoresuscitation”. To avoid duplication, each database was independently reviewed by two members of our team and duplicated records were excluded, as well as review articles. In the original reports we searched cases of Lazarus syndrome. From all records we have found that (49,316) only 1655 entries met the criteria for inclusion in the initial analysis. After further exclusion of 1494 reports, we got 65 publications in which 81 cases of Lazarus syndrome were described. Then we searched the group of patients older than 60 years of age and finally we chose 48 patients for further analysis. A flowchart of case selection is presented in Figure 1.

Table 1 presents all cases of Lazarus phenomenon aged 60 years and older who were included in the study. All data relating to cardiac arrest, the course of resuscitation and the onset of LP that could be gathered from the literature are presented here. The individuals were divided in two subgroups (aged 79–60 years and aged ≥80 years).

The statistical analysis was performed in Statistica 13 (TIBCO Software Inc., Palo Alto, CA, USA). Statistical significance was defined as *p* < 0.05. The Shapiro–Wilk test was used to assess conformity with a normal distribution. Numerical variables were presented as median with interquartile range (IQR). In order to compare the variables between the two groups, the *t*-test or the Cochran–Cox test was calculated due to compliance with the normal distribution. The Mann–Whitney test was calculated in case of lack of compliance with the normal distribution. Categorical variables were reported as absolute numbers and percentages and compared using Pearson’s chi-squared test or Fisher exact test. Due to the lack of compliance with the normal distribution the association between numerical variables was assessed by Spearman’s correlation. Taking into account the data bias in these publications, the data that were collected (disease, location of event, gender, age, etc.) are variables in which errors are unlikely to occur. To avoid additional bias, we omitted this information (treated it as missing data) when the data were imprecise (mainly in the area of survival time).

Additionally, for these analyses, due to the small sample size, instead of relying solely on the *p*-value, we also calculated an effect size (Cohen’s d or Pearson’s C), which quantifies the magnitude of the difference between groups. Because we looked at the effect size, not just the *p*-value, multiple comparisons (which modify the *p*-value) were not performed.

Given the small size and heterogeneity of the group, as well as the absence of certain key variables, the results obtained should be treated with caution as preliminary findings.

Doing a statistical analysis, we have taken into account: the origin of the diseases before cardiac arrest (CA) which could be of cardiac—C—or non-cardiac—NC—origin, place of CA (in hospital—IH—or out of hospital—OH), age and gender of the patients, duration of CPR, rhythm during CPR just before occurrence of the LP, time from stopping CPR to occurrence of the LP, return of consciousness and duration of survival.

As for missing data, it should be emphasised that they were not related to a specific publication, but occurred in all publications and in most of the analysed variables. Specific missing data are presented in Table 2.

## 3. Results

We have included in the study 48 individuals. The results are presented in Table 3.

The causes of cardiac arrest were divided almost equally between cardiac and non-cardiac causes (47.6% and 52.3% respectively). Cardiac arrest occurred equally in the IH and OH, each accounting for 50%. There were 22 women and 26 men. The ECG rhythm during cardiopulmonary resuscitation prior to the onset of LP was as follows: A—58.7%, PEA—37% and VF—4.3% respectively, confirming that non-shockable rhythms predominate. In 16 out of 37 cases where such data were reported, a return to consciousness was confirmed, representing 43.2%. The duration of cardiopulmonary resuscitation varied—from a minimum of 6 min to 90 min. Time of cessation of CPR to the occurrence of the LP was also different—from a minimum of 2 min to even 180 min. Survival time following the occurrence of the Lazarus phenomenon varied considerably, ranging from a few minutes to 90 days; however, the survival time of patients who were still alive at the time of reporting (7 patients) was not included.

For further analysis we have divided senior individuals according to age into two subgroups: No 1 (60–79 years old), No 2 (≥80 years old). In Group No 1, we have analysed 26 cases (16 men, 10 women). In Group No 2 we have analysed 22 cases (10 men and 12 women). An analysis was carried out based on the available data from the literature regarding the presented cases of LP, and it should be treated with great caution due to the lack of all the data required for analysis.

Detailed description and comparison of groups based on statistical analysis

(1) The comparison between two groups: No 1 (60–79), No 2 (≥80).
  Comparing two groups and taking into account the following categorical variables—causes of CA (of cardiac versus non-cardiac origin), place of CA (IH and OH), gender, ECG rhythms before occurrence of LP, and return of consciousness—no relationship was found taking into account simultaneously, in addition to *p*, the contingency coefficient. When continuous variables were analysed, (duration of CPR, time of cessation of CPR to the occurrence of the Lazarus phenomenon and time of survival), only a slight difference (d-Cohen = 0.25) was noticed in time of survival. In group No. 1 (aged 60–79), patients lived longer.
(2) The analysis of Group No 1 (60–79 years of age).
  Analysing Group, No 1 and taking into account the categorical variables, causes of CA (of cardiac versus non-cardiac origin), place of CA (IH and OH), gender, ECG rhythms before occurrence of LP, and return of consciousness, no relationship was found, taking into account simultaneously, in addition to *p*, the contingency coefficient. The relationship between return of consciousness and place of CA was found (*p* = 0.03). Four patients recovered when CA occurred IH and one patient in out-of-hospital CA. A total of 5 patients out of 13 analysed. A relationship between return of consciousness and age of the patients was found (*p* = 0.01). In Group No 1 (aged 60–79), more patients regained consciousness than in the group aged 80 and over. It should be noted here that this involved 9 out of 26 patients, and the available data on this matter related to only 18 patients.
(3) The analysis of Group No 2 (≥80 years of age).
  Analysing Group No 2 and taking into account the categorical variables, causes of CA (of cardiac versus non-cardiac origin), place of CA (IH and OH), gender, ECG rhythms before occurrence of LP, and return of consciousness, no relationship was found, taking into account simultaneously, in addition to *p*, the contingency coefficient, except for ECG rhythm before LP in correlation to causes of CA (*p* = 0.03). Eleven individuals presented asystole (9—of C origin, 2 of NC), 5 presented PEA (1—of C origin, 4 of NC). When continuous variables were analysed (duration of CPR, time of cessation of CPR to the occurrence of the Lazarus phenomenon and time of survival), only a slight difference (d-Cohen = 0.27) was noticed in time of cessation of CPR to the occurrence of the Lazarus phenomenon in correlation to gender.

In this group of patients, a correlation between duration of CPR and time of survival was found. The longer the time of CPR, the longer the patients’ survival time (Rs = 0.60).

## 4. Discussion

At the current stage of knowledge, the causes of Lazarus phenomenon are only presumed, but some of them may include hyperventilation, alkalosis, auto-PEEP and delayed action of drugs. Unfortunately, there is no data in the literature on the quality of cardiopulmonary resuscitation and the likelihood of hyperventilation or alkalosis, as well as other alleged causes of the LP. Therefore, they could not be examined. When analysing the available data, we encountered a problem of missing data, which may have affected the results.

In our study, the causes of cardiac arrest were distributed almost equally between cardiac and non-cardiac causes. Cardiac arrest occurred with equal frequency in the IH group and the OH group. The ECG rhythm during cardiopulmonary resuscitation prior to the onset of LP was as follows, A—58.7%, PEA—37% and VF—4.3%, confirming the predominance of non-shockable rhythms, which is consistent with data from the literature indicating that older people who are in cardiac arrest more often present non-shockable rhythms such as PEA and asystole [54,55]. In a German study, retrospective analysis of out-of-hospital resuscitation patients over 80 years of age (n = 578) showed that 86,1% of them initially had asystole—53.2%—and PEA—32.9%. What is interesting, in the surviving group of patients, is that 60% presented non-shockable rhythm (22.9% asystole and 37.1% PEA) [55].

Non-shockable rhythms are more common in patients with Lazarus phenomenon—cardiac causes are less common in patients with asystole or PEA [56,57].

At present, the missing data are not significant, as the cause of cardiac arrest was not specified in 6 out of 48 cases, and data relating to the ECG rhythm analysis were missing in 4 out of 48 cases.

Discussing the causes of Lazarus phenomenon in elder people, we have to take into account the ageing process and progressive organ changes. In elder people the prevalence of ECG abnormalities is common and according to Moplaschi M. et al. even three times higher in patients over 85 years than in younger patients (65–69 years old) [58]. These abnormalities include: first-degree atrioventricular block, right and left bundle branch block, atrial fibrillation (which is more common as age increases), ventricular ectopic contractions (occurs from 76 to 96%), major ST-T wave alterations.

In old people the physiology of circulatory system differs from younger subjects. During ageing, calcification of the cardiac skeleton is common, as well as decline of cardiac myocytes and an increase in elastic and collagenous tissue. Also, a progressive loss of pacemaker cells within the sinus node occurs, especially over 75 years of age. Due to a partial or complete separation of the sinoatrial node from surrounding musculature caused by accumulation of adipose tissue, action of potential is prolonged and autonomic response is reduced [59]. These cardiac changes in older people may contribute to the occurrence of the Lazarus phenomenon. Additionally, in older people, metabolism is slower, and one potential reason for the Lazarus phenomenon is slower delivery of drugs to the central circulation and ultimately delayed drug action, which should be taken into account. In our study the time to the occurrence of Lazarus phenomenon in analysed subjects has been extended even to 20 min and more. In the analysed younger subgroup of patients (79–60 years old) the time from the end of CPR to the occurrence of Lazarus phenomenon was longer compared with the older subgroup (≥80 years of age). It necessitates further study for explanation. The greatest challenge is to determine the duration of patient monitoring from the moment of death. The longest time between cessation of CPR and the return of circulatory function occurs in the case of asystole, which should make us even more sensitive to extending post-mortem observation. Because statistics show that the longer CPR is continued, the later Lazarus phenomenon may occur, post-mortem monitoring should be extended in these cases.

In the study, only in the group ≥80 years old a correlation between duration of CPR and survival in days was found, which should be handled with great care. One might suppose that the longer the time of CPR, the longer the survival in days, and this requires further data to be collected.

This result surprisingly indicates the possibility of recovery of very old individuals when cardiopulmonary resuscitation was performed for longer. It can be explained by slower metabolism in this group of patients and relative overdose due to a decrease in the functional reserve of organs.

The group of patients over 60 years of age is characterised by a higher frequency of comorbidities, polypharmacy, and above all, a lower physiological reserve. The most important aspect of the ageing process is the gradual reduction in functional units, such as nephrons in the kidneys and hepatocytes in the liver. Age-related changes in organ function affect drug distribution and elimination pathways, leading to prolonged drug action and an increased risk of drug accumulation. Geriatric patients are more susceptible to drug toxicity and adverse drug reactions due to elevated serum concentrations. Reduced sensitivity of β-1 and β-2 adrenergic receptors in the heart is observed, leading to a weakened response to β-agonists. Alterations in homeostatic mechanisms, such as attenuation of reflex tachycardia and decreased baroreceptor function, and impaired autoregulation in critical organs (brain, heart, and kidneys) increase the risks associated with “relative” overdose [60,61].

Other alleged causes of Lazarus phenomenon (alkalosis, hyperkalaemia, metabolic disorders) are difficult to explain in the context of generating spontaneous recovery of cardiac function because they are considered as reversible causes of cardiac arrest. According to the Guidelines of Resuscitation during cardiopulmonary resuscitation, reversible causes of cardiac arrest should be found and, if confirmed, treated at once.

## 5. Conclusions

The descriptive analysis was based on data available in the literature, drawing on 48 cases. Based on these cases, we can suspect the following conclusions. In older people, even those of very advanced age, the Lazarus phenomenon may occur following resuscitation or even if resuscitation is not attempted. It may even occur after a very prolonged resuscitation. Based on our analysis of the available literature on LP case reports, we found no correlations when taking into account causes of CA, place of CA, gender, ECG rhythms before occurrence of LP, return of consciousness, time of CPR, time of cessation of CPR to the occurrence of the P and time of survival, so, at this stage, we are unable to determine the exact cause of LP in older people, partly due to the small sample size and the lack of all the necessary data. The causes of LP probably lie outside the analysed data. Accurate reporting is required, including data such as: CPR time points with details of medication administered during CPR, airway management, quality of ventilation, and laboratory tests (blood gas analysis, electrolyte levels, complete blood count) in order to analyse the suggested causes of LP.

Future research should include disseminating knowledge, understanding the pathophysiology of this phenomenon, and implementing preventive measures (e.g., prolonged monitoring after death is confirmed). The duration of prolonged monitoring was suggested based on information from the literature. Return of circulation occurred within 20 min in 90% of the cases we analysed. However, further case reports and analyses are needed to determine an appropriate and long-term follow-up period.

This phenomenon has implications not only for cardiopulmonary resuscitation procedures but also for ethical issues. It would be worthwhile to include information on this topic in the “Ethics of Resuscitation” section/subsection of the ERC Guidelines.

As a potential cause of Lazarus phenomenon, age-related changes should be taken into account, such as progressive organ changes, slower metabolism, changes in drug distribution and elimination pathways, leading to prolonged drug action and an increased risk of drug accumulation.

## 6. Limitations of the Study

The study was based on the cases described in the literature; therefore, unfortunately, there was no information available on the current medical history, comorbidities, previous injuries or lifestyle of the subjects and, furthermore, there was a lack of information regarding the quality of the CPR performed, the timing of medication administration, airway management, ventilation, and laboratory tests, so they were not taken into account. The scarcity and quality of available data is the main limitation of the study. The lack of data makes it difficult to draw clear conclusions. The group of seniors who developed LP that was analysed was not very large, as presumably not all cases of LP are reported in the literature.

## Figures and Tables

**Table 1:** Cases of Lazarus phenomenon among patients aged 60 years and older included in the study (divided in two subgroups).

Year of Publication Number of References	Diseases Before CA and Place of CA (If Known)	Age (Years)	Sex	The Duration of CPR (in min)	ECG Rhythm Before Occurrence of the LP	Occurrence of the LP After Cessation of CPR (min)	Return of Consciousness	Survival (in Days)
Cases of Lazarus phenomenon among patients aged 60–79 years								
1982 [1]	C–MI	67	M	20	A	<9	ND	15
	C–MI	68	F	ND	A	20	Yes	90
1991 [4]	NC, Co, IH	64	M	20	PEA	15	ND	<1
1991 [14]	NC, OH	75	F	20	A	5	No	<1
1993 [2]	NC	75	M	23	A	5	No	<9
1994 [15]	C, Co, IH	70	M	34	A	8	Yes	21
1996 [16]	C, MI, IH	66	M	30	VF	<5	Yes	S
1998 [17]	C, MI, IH	67	F	43	A	5	Yes	9
1999 [18]	NC, Co, IH	76	M	30	A	5	No	1
2001 [19]	NC, Co, IH	66	M	18	A	10	Yes	13
2002 [20]	NC, Co, OH	65	M	35	A	20	No	5
2005 [21]	NC	63	F	12	A	10	Yes	12
2006 [22]	C, MI, OH	78	M	25	A	<9	No	<1
2012 [23]	C, MI, OH	65	M	55	A	40	No	13
2013 [24]	NC, IH	62	M	40	PEA	5	ND	<2
2015 [25]	NK, Co	67	M	47	PEA	5	Yes	1
2016 [26]	NC	69	F	25	PEA	10	ND	S
2017 [27]	NC, OH	69	M	40	A	180	No	10
2017 [28]	NC	63	M	12	PEA	3	Yes	>1
	NC	61	F	18	PEA	8	No	<1
2017 [29]	C, MI, Co	66	M	45	PEA	5	Yes	9
2020 [30]	NC, OH	79	F	9	PEA	14	No	<1
2021 [31]	NC, Co, IH	79	F	10	A	20	ND	14
2021 [32]	IH	66	F	32	PEA	5	ND	ND
2023 [33]	C, OH	67	F	60	PEA	60	Yes	S
2025 [34]	C, OH	78	M	40	VF	3	ND	<1
Cases of Lazarus phenomenon among patients aged ≥80 years								
1982 [35]	C, MI	80	M	20	A	5	Yes	35
1982 [1]	C, MI	84	M	10	A	5	No	1
1993 [36]	NC, Co, IH	87	F	>15	ND	ND	ND	12
1998 [37]	NC, Co	80	M	30	PEA	5	No	2
2001 [38]	NC, Co, IH	93	F	6	ND	5	No	ND
2003 [39]	NC, Co, IH	81	M	25	A	2	Yes	31
2004 [40]	C, MI, OH	81	F	13	A	<9	No	<1
2004 [41]	NC, Co, IH	94	F	40	PEA	3	ND	21
2005 [42]	C, MI, OH	83	F	17	A	33	No	<1
2006 [43]	C, OH	83	M	60	PEA	7	Yes	ND
2007 [44]	NC, Co, IH	85	M	DNR	PEA	6	No	2
2010 [45]	NC, Co, OH	84	M	15	PEA	5	Yes	S
2011 [46]	NC, Co, OH	83	M	90	A	10	No	12
2013 [47]	NC, Co, IH	89	F	18	A	5	No	<16
2017 [28]	NK	97	F	16	A	3	No	<1
	NK	91	F	16	PEA	3	No	<1
2018 [48]	C, Co	97	M	DNR	A	ND	No	<1
2019 [49]	C	86	F	DNR	A	4	Yes	S
2019 [50]	C, Co, OH	86	F	40	A	15	No	3
2023 [51]	NC, Co	88	M	20	PEA	60	No	<1
2025 [52]	C, OH	94	F	DNR	A	10	Yes	S
2025 [53]	C, Co, OH	88	F	DNR	A	<9	Yes	S

Abbreviations: CA—cardiac arrest, C—cardiac origin, NC—non-cardiac origin, IH—in-hospital CA, OH—out-of-hospital CA, ND—no data, MI—myocardial infarction, Co—comorbidities, A—asystole, PEA—Pulseless Electrical Activity, VF—ventricular fibrillation, DNR—do not resuscitate, S—survived.

**Table 2:** Number of missing data by analysed categories.

Analysed Categories Among 48 Cases	Number of Missing Data
Causes of cardiac arrest (C or NC)	6
Place of cardiac arrest (IH or OH)	16
Time of CPR	7
Rhythm during CPR before occurrence of the LP	4
Time from cessation of cardiopulmonary resuscitation to the occurrence of the LP	7
Return of consciousness	8
Survival	13

C—cardiac, NC—non-cardiac, IH—in hospital, OH—out of hospital, CPR—cardiopulmonary resuscitation, LP—Lazarus phenomenon.

**Table 3:** Results of the data analysis, including statistical findings, in two patient subgroups based on the available data.

Analysed Data	Group No 1 (n = 26)	Group No 2 (n = 22)
Causes of cardiac arrest	C—10, NC—14	C—10, NC—8
Place of CA	IH—9, OH—8	IH—7, OH—8
Gender	F—10, M—16	F—12, M—10
ECG rhythm during CPR before occurrence of LP	A—14, PEA—10, VF—2	A—13, PEA—7
Return of consciousness	Y—9, N—9	Y—7, N—13
The duration of CPR (in minutes)	n = 25 median—30.00 min-9.00 max-60.00 lower quartile—20.00 upper quartile—40.00	n = 16 median—19.00 min-6.00 max-90.00 lower quartile—15.50 upper quartile—35.00
Time of cessation of CPR to the occurrence of the LP (in minutes)	n = 23 median—8.00 min-3.00 max-180.00 lower quartile—5.00 upper quartile—20.00	n = 18 median—5.00 min-2.00 max-60.00 lower quartile—4.00 upper quartile—10.00
Time of survival (in days)	n = 19 median—9.00 min-0.002 max-90.00 lower quartile—0.79 upper quartile—13.00	n = 16 median—1.50 min-0.001 max-35.00 lower quartile—0.21 upper quartile—12.00

C—cardiac, NC—non-cardiac, IH—in hospital, OH—out of hospital, CPR—cardiopulmonary resuscitation, LP—Lazarus phenomenon, F—female, M—men, Y—yes, N—no, A—asystole, PEA—Pulseless Electrical Activity.

## Data Availability

No new data were created or analyzed in this study. Data sharing is not applicable to this article.
